# JMM-TGT: Self-supervised 3D action recognition through joint motion masking and topology-guided transformer

**DOI:** 10.1371/journal.pone.0338008

**Published:** 2025-12-30

**Authors:** Han Wen, Guangping Zeng, Qingchuan Zhang, Zihan Li, Mengyang Zhu

**Affiliations:** 1 School of Computer and Communication Engineering, University of Science and Technology Beijing, Beijing, China; 2 School of Computer and Artificial Intelligence, Beijing Technology and Business University, Beijing, China; Southeast University, CHINA

## Abstract

In the field of 3D skeleton action recognition, research on self-supervised learning methods has primarily focused on spatio-temporal feature modeling. However, these methods rely heavily on modeling single motion features, which limits their ability to capture subtle motion variations and complex spatio-temporal relationships. This is a direct result of the fact that understanding the model of the action remains incomplete. To address the above-mentioned issue, this paper proposes the Joint Motion Masking with Topology-Guided Transformer model (JMM-TGT) for action recognition. First, the Joint Motion Masking strategy is applied to enhance the ability of the model to perceive subtle joint movements. This method can generate masking probabilities by combining the differences and similarities in joint motion, thereby guiding the selection of joints to be masked at each time step. Meanwhile, in the transformer-based encoder module, the topological relationship between joints is introduced to adjust the attention mechanism, allowing the model to capture spatio-temporal dependencies and better understand the complex dynamic patterns of joint motion. To verify the performance of the JMM-TGT model, we conducted comparison experiments between it and mainstream action recognition models. Experiments demonstrate that the proposed JMM-TGT achieves performance improvements ranging from 1.5% to 7.9% under different evaluation settings on the NTU RGB+D 60, NTU RGB+D 120, and PUK-MMD datasets.

## 1 Introduction

Action recognition is the task of identifying and categorizing specific actions within videos for classification purposes [1]. It is widely applied in various fields such as virtual reality [2], autonomous driving [3], and video surveillance [4]. Advances in pose estimation algorithms [5] have simplified the acquisition of 3D skeleton data, which is also more resistant to interference. These factors have made 3D skeleton-based action recognition a popular research topic in recent years. Driven by graph convolutional networks [6] and transformer [7], numerous supervised learning methods have emerged, achieving excellent performance due to their reliance on large amounts of labeled data. However, the increasing cost of acquiring labeled data makes it difficult to rely solely on supervised learning. As a result, using unlabeled data for training has become increasingly important. Semi-supervised learning [8] with a small amount of annotated data and unsupervised learning based on unlabeled data have gradually attracted attention. However, these methods still face limitations in terms of accuracy and generalization. Therefore, self-supervised learning becomes a promising solution in the field of action recognition. This method learns through the intrinsic structure of the data itself, which not only significantly reduces the dependence on labeled data, but improves the learning ability of the model on large-scale unlabeled data.

The introduction of the Multi-Layer Perceptron (MLP) [9] has advanced the application of self-supervised learning in spatio-temporal modeling. This type of modeling captures complex dependencies across both space and time in videos, helping the model better understand the spatial and temporal structure of actions. Spatio-temporal feature modeling aims to capture the complex dependencies across both space and time in videos, enabling the model to better understand the temporal and spatial structure of actions. Currently, research focuses on efficiently extracting spatio-temporal features to improve the accuracy and robustness of action recognition models, particularly in complex environments. Given the impressive performance of Graph Convolutional Network (GCN) and transformer in action recognition tasks, researchers have explored combining these Networks with others. The integration of the GCN and Contrastive Learning, particularly Cross-Stream Contrastive Learning [10], has successfully reduced the reliance on labeled data as well as introduced new perspectives for spatio-temporal feature modeling. Furthermore, Contrastive Learning with Cross-Part Bidirectional Distillation [11] enhances the understanding of action in the model by applying bidirectional distillation between different parts of joints and the skeleton. Moreover, the combination of masking strategies has shown promising results. For example, the Spatial-Temporal Masked Autoencoder framework (SkeletonMAE) [12] demonstrates its unique advantages of combining self-supervised learning with masking strategies by randomly masking certain joints or frames and using transformers for prediction. To improve model performance in complex action recognition tasks, spatio-temporal feature modeling has increasingly focused on capturing relative motion. The approach of using similarity modeling between local and global skeleton sequences [13] has laid a solid foundation for subsequent research. This method effectively captures richer spatio-temporal relationships between actions across different skeleton parts. Finally, the Relative Temporal Velocity Contrastive Learning framework for skeleton action Represetation (RVTCLR) [14] highlights the importance of relative motion modeling in ecognizing subtle movement differences, specially for fine-grained and cross-view action recognition.

Although existing spatio-temporal feature modeling approaches have advanced 3D skeleton action recognition to some extent, they still struggle with capturing subtle motion variations and complex spatio-temporal dependencies. Current models tend to overly rely on single motion feature modeling [12], which makes it difficult to capture global dynamic changes caused by small motion differences, thereby limiting the ability of model to fully understand actions. Moreover, these models often overlook the structured information in skeleton data [12–14], preventing them from effectively learning and utilizing the spatio-temporal dependencies between joints in complex actions. The oversight affects both action recognition accuracy and the generalization ability of model.

**Fig 1:**
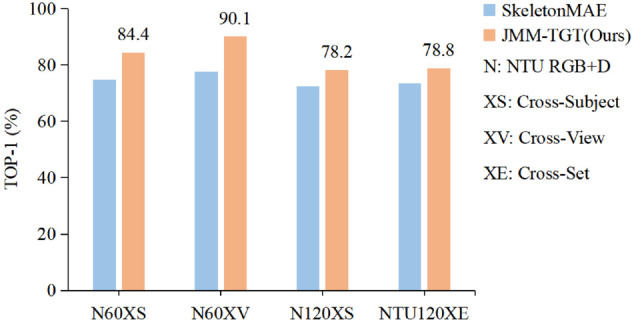
A comparison of the proposed method with SkeletonMAE(ICMEW 23) [12], using linear probing evaluation protocol.

To address these defects, this paper proposes the Joint Motion Masking with Topology-Guided Transformer model (JMM-TGT) for 3D skeleton action recognition. The core idea of this model is to enhance the fine-grained modeling of complex spatio-temporal dependencies by incorporating joint motion difference, similarity information, and inter-joint topological relationships. To be specific, the main contributions of this paper are as follows:

1. Proposed a joint motion masking strategy, JMM. Integrates the differences and similarities in joint motion across adjacent frames as priors, employing an adaptive skeleton masking approach to better focus on semantically rich temporal regions. The motion similarity information enhances the ability of model to perceive subtle joint movements. This method achieves the fusion of global and local motion features of joints, effectively overcoming the limitations of relying on a single motion modeling approach.
2. Designed a topology-guided Transformer, TGT. We innovatively introduce topological relationship between joints into the attention mechanism, which not only captures spatial dependencies between joints but also ensures the effective learning of temporal details, enhancing the focus on key joint motion variations, and realizes the understanding of complex actions in the model.
3. To validate the effectiveness of the model, experiments are conducted on NTU RGB+D 60, NTU RGB+D 120, and PUK-MMD datasets. The accuracy reached 84.4% and 90.1% under the X-Sub and X-View protocols on the NTU-60, respectively, and 78.2% and 78.8% under the X-Sub and X-Set protocols on the NTU-120, such as Fig 1. Additionally, transfer learning on the PKU-II achieved an accuracy of 73.0%. These results validate the effectiveness of JMM-TGT and its excellent transfer ability.

## 2 Related works

According to different training methods, action recognition approaches can be divided into two categories: supervised and unsupervised-based learning methods, as well as self-supervised learning-based methods. As shown in Table 1, summarizes the main self-supervised learning related works.

**Table 1:** The summary of related work on self-supervised learning methods.

Contrastive Learning	Feature extraction
CSCLR [10]	Relies on high similarity positive samples.
Contrastive Learning with Cross-Part Bidirectional Distillation [11]	Limited consideration of the environment or contextual information.
Skeleton-logoCLR [13]	Using the similarity of global and local skeleton sequences.
**Spatio-temporal feature modeling**	**Feature extraction**
SkeletonMAE [12]	Uses masking strategy and transformer with single motion feature modeling.
RVTCLR [14]	Relative motion captures the fine-grained differences in actions.

### 2.1 Supervised and unsupervised action recognition

Supervised learning methods train models using labeled data, enabling them to predict or classify new and unlabeled data. Due to the spatio-temporal nature of skeleton data, Graph Convolutional Network (GCN) has become widely used in action recognition. Yan S et al. [6] introduce the Spatial-Temporal Graph Convolutional Network (ST-GCN), which learns spatial and temporal patterns from data, overcoming the limitations of traditional methods. Cheng K et al. [15] propose Shift-GCN to address high computational costs and rigid receptive fields of traditional GCN. The Select-Assemble-Normalize Graph Convolutional Network (SAN-GCN) for improved feature modeling is introduced by Tian H et al. [16]. Jang S et al. [17] develope Multi-Scale Structural Graph Convolutional Network (MSS-GCN) to enhance high-dimensional information use and multi-scale aggregation. Zhang Y et al. [18] introduce Lightweight Graph Convolutional Network (LGCN) to reduce computational complexity for real-time applications. The Hybrid Network of Skeleton Guidance and Supervision (SGS-HN) is proposed by Ren Z et al. [19], improving multimodal feature learning and inter-modal alignment. Xu J et al. [20] create an end-to-end skeleton model with an Out-of-Distribution detection mechanism for better adaptability. To overcome the challenges posed by perspective changes and varying speeds, Aouaidjia K et al. [21] propose Spatio-Temporal Invariant Descriptors (STID). Recent studies have explored the use of Generative Adversarial Networks (GANs) [22] in action recognition, improving data diversity and model robustness.

To improve the ability of the model to capture both temporal and spatial features, the transformer has become an essential component. Yang G et al. [7] introduce the Spatial-Temporal Attention Temporal Segment Network (STA-TSN), which combines an attention mechanism to improve sensitivity to dynamic changes and accurately capture key features in time-series data. Multi-AxisFormer (MAFormer) is proposed by Huang H et al. [23] to address limitations in integrating hierarchical information and handling ultra-long time series. In addition, the cross-attention [24], dynamic attention [25], and multi-scale attention mechanisms [26] effectively utilize information from multiple perspectives, enhancing the capabilities of model. It is worth noting that Xu Z et al. [27] propose Satiotemporal Decoupling Attention Transformer (SDAT) to address the issue of spatio-temporal interaction in complex action patterns.

Supervised learning methods require large amounts of labeled data, which is expensive and labor-intensive. Additionally, these methods struggle to generalize to new scenarios and cannot effectively utilize the vast amount of available unlabeled data. To break through these limitations, semi-supervised and unsupervised learning methods have gained attention. Semi-supervised learning combines a small amount of labeled data with a large volume of unlabeled data, with models like Graph Representation Alignment (GRA) [8] and Dual-Stream Cross-Fusion with Class-Aware Memory Bank (DSCF-CAMB) [28] addressing challenges in category modeling and feature alignment. However, semi-supervised learning still rely heavily on the quality of unlabeled data and often involves high model complexity. In contrast, unsupervised learning eliminates the need for labeled data, significantly reducing labeling costs. Lin L et al. [29] propose a comparative learning framework based on Actionlet to enhance feature discriminability, while RDCL [30] captures complex joint relationships. He Z et al. [31] introduce Multi-Domain Decoupling Representation Modeling to improve cross-domain generalization, and Liu Z et al. [32] develop a multi-view action recognition method. Lin L et al. [33] propose an idempotent unsupervised representation learning method to stabilize skeleton data representations. However, interpreting and evaluating unsupervised methods remains challenging, limiting their development.

### 2.2 Self-supervised learning for action recognition

Current research on self-supervised learning methods can be divided into spatio-temporal feature modeling, relative motion modeling. In spatio-temporal feature modeling, Jin Z et al. [34] propose the Self-supervised Spatial-Temporal Representation Learning (SSRL), which improves model performance in complex dynamic environments by jointly modeling spatial and temporal features. Yao S et al. [35] introduce a GCN-based approach for recognizing martial arts leg poses in multimodal robots. Due to the inherent nature of self-supervised learning, masking strategies are particularly effective as a complementary method. These strategies force the model to learn useful feature representations by masking parts of the data, requiring the model to perform reconstruction. SkeletonMAE is proposed by Wu W et al. [12], using spatial-temporal masking strategies where joints and frames are randomly masked based on a predefined percentage. This framework leverages transformer to predict the masked joints or frames, effectively capturing dynamic features. Similarly, the Spatiotemporal Clues Disentanglement Network (SCD-Net) proposed by Wu C et al. [36] decouples spatio-temporal information through masking. It applies spatial bootstrap masks based on skeleton structure and temporal bootstrap masks on small portions of the time series. In addition, the puzzle problem training method [37] disrupts action sequences to teach the model to learn the correct order. Given the effectiveness of masking strategies, this paper adopts them as a core method.

All of the above methods rely on single motion feature modeling and struggle with the challenge of insufficiently capturing fine-grained motion patterns. Thus, relative motion modeling becomes crucial. Contrastive learning is a popular method for learning efficient representations by comparing similarities and differences between samples. Common methods include Cross-Stream Contrastive Learning [10], the Dual Min-Max method from game theory [38], and the Extremely Enhanced Contrastive Learning method [39]. Hu J et al. [13] improve model performance by combining global and local similarity modeling to capture fine-grained features. Futhermore, spatio-temporal information integration in relative motion modeling has been explored to improve adaptability in complex environments, with approaches like spatio-temporal comparative clustering [40] and multi-scale motion comparison learning [41]. The Cross-Part Bidirectional Distillation method is proposed by Yang H et al. [11], which focuses on learning features from different skeleton parts and emphasizes local motion. Liu R et al. [42] propose a semantic representation-guided contrastive learning method, enhancing the understanding of action details. However, contrastive learning performance depends heavily on the quality of negative sample selection. Poor negative sample selection can result in ineffective model training. Moreover, the difficulty in clearly defining positive and negative samples in some tasks impacts the training effectiveness. To address these issues, Zhu Y et al. [14] introduce the RVTCLR model, which focuses on capturing rhythmic variations and relative temporal relationships between movements. This method has shown effectiveness in capturing subtle movement differences. Based on this, this paper models motion features by calculating joint motion differences and similarities across adjacent frames.

In summary, existing self-supervised learning methods mainly focus on contrastive learning, and the masking strategies still need further refinement. Spatio-temporal feature modeling typically relies on either absolute or relative motion alone, lacking integrated modeling of both local and global features. On the other hand, in transformer-based skeletal structures, the model often overlooks subtle differences in local motion and the spatio-temporal context, leading to poor performance in understanding complex actions. Therefore, this paper proposes the JMM-TGT, which captures both local and global features by leveraging the differences and similarities in joint motion, and introduces joint topological relationships as priors to enhance the ability of model to understand complex actions.

## 3 Methodology

**Fig 2:**
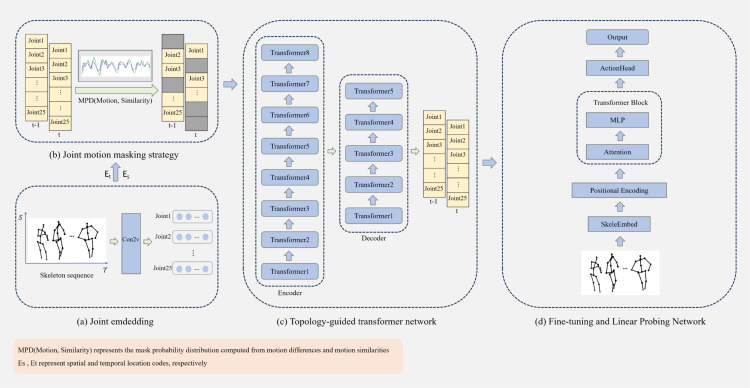
Joint motion masking with topology-guided transformer model for action recognition.

The overall framework of this paper is shown in Fig 2. The model consists of three core components: joint embeddings, joint motion masking strategy, and topology-guided transformer. The first stage involves a base embedding layer that converts raw skeleton data into a valid embedding representation. In the second stage, the model determines the masked joints by combining differences and similarities in joint motion across adjacent frames. The third stage is the pre-training phase, which utilizes transformers for encoding and decoding. In this stage, this paper introduces the topological relationships between joints as prior into the attention layer, while also incorporating positional and temporal encoding to predict the motion sequence over time.

### 3.1 Joint motion masking strategy

In this study, the joint motion masking strategy (JMM) generates masking probabilities by integrating the differences and similarities in joint motion between adjacent frames. Based on these probabilities, the model dynamically selects which joints to mask at each time step during training. Specifically, joint motion difference focuses on absolute movement changes at each time step, helping the model capture significant motion variations. For instance, when a joint shows a large motion difference, the model can quickly identify this change. Meanwhile, joint motion similarity emphasizes how small local movements can cause larger changes in the global motion pattern, i.e., relative motion. This helps the model detect subtle changes that impact the overall action. By combining these two aspects, the model is better able to focus on fine-grained motion changes, improving its ability to recognize complex actions. This strategy enhances the ability of model to perceive both global and local motion features, overcoming the limitations of traditional methods that rely solely on single-motion features.

**Fig 3:**
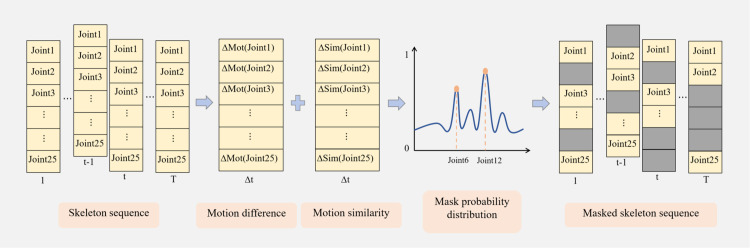
Joint motion masking strategy for action recognition.

As shown in Fig 3, the implementation process of the JMM is outlined in detail. First, the formulas for calculating joint motion difference and similarity are presented, followed by an explanation of how to generate the final motion information by combining these differences and similarities in joint motion. Next, the application of the masking strategy in the model is fully outlined, including the calculation of the masking probability and the process of generating the masking matrix.

#### 3.1.1 Joint motion difference.

In order to quantify the change in motion of the joint at each time step, denoted as $x_{\text{mot }}\in {\mathbb{R}}^{1\times T\times V\times C}$, the absolute value of the positional difference is calculated.

$${x_{\text{mot}}}_{v}^{t}(c)=\left|x_{v}^{t}(c)-x_{v}^{t-1}(c)\right|,\;t\in T,v\in V,c\in C$$ (1)

Here,

- *x* = Skeleton sequence.
- *T* = Number of time frames.
- *V* = Number of joints.
- *C* = Number of channels.

where $x_{t}^{v}(c)$ is the difference in motion at joint *v* in the *c* dimension at time step *t*, and the ${x_{\text{mot}}}_{v}^{t}(c)$ is the difference in motion at joint *v* in the *c* dimension at time step *t*.

Since the joint motion difference between adjacent frames are calculated, the time step is set to 1. As the first frame lacks a previous frame, zero padding is applied in the subsequent calculations. The calculation of joint motion similarity in the following is the same.

#### 3.1.2 Motion similarity.

To further optimize the masking strategy, the similarity of each joint at adjacent time steps is also calculated, denoted as $x_{\text{sim }}\in {\mathbb{R}}^{1\times T\times V\times C}$. The similarity measures the trend of motion change of joint *v* in the *c* dimension between the current time step *t*, and the previous time step *t*–1.

$${x_{\text{sim }}}_{v}^{t}(c)=\frac{\left|\sum_{t}\left(x_{v}^{t}(c)-\mu_{x_{v}^{t}}(c)\right)\circ \left(x_{v}^{t-1}(c)-\mu_{x_{v}^{t-1}}(c)\right)\right|}{\left(\sqrt{\sum_{t}{\left(x_{v}^{t}(c)-\mu_{x_{v}^{t}}(c)\right)}^{2}}\cdot \sqrt{\sum_{t}{\left(x_{v}^{t-1}(c)-\mu_{x_{v}^{t-1}}(c)\right)}^{2}}\right)+1e-10}$$ (2)

Here,

- 1*e*–10 = Prevent division by zero.

where $\mu_{x_{v}^{t}}(c)$ and $\mu_{x_{v}^{t-1}}(c)$ are the mean values of joint *v* in dimension *c* at time steps *t* and *t*–1, respectively, and the similarity ${x_{\text{sim }}}_{v}^{t}(c)$ is the ratio of the numerator to the denominator, which measures the similarity of the motion pattern between the current frame and the previous frame. If the similarity is close to 1, it means that the motion changes between the two time steps are very similar.

Moreover, The numerator denotes the result of the element-by-element multiplication of the difference vectors of the joints between the current and previous frames, indicating the magnitude of change for each joint in each dimension. The denominator is the Euclidean distance of the change between the two time steps, which measures the overall motion of each joint between the current and previous frames.

#### 3.1.3 Final motion information.

To ensure the model can choose the retained time step precisely in the masking phase, the joint motion difference and similarity are combined to get the final motion information, denoted as $x_{\text{fin\_mot}}\in {\mathbb{R}}^{1\times T\times V\times C}$.

$$x_{\text{dec }}=\mathrm{Decoder}\left(x_{\text{enc }}\right)$$ (3)

where ${x_{\text{fin\_mot}}}_{v}^{t}(c)$ denotes the final motion information of joint *v* in the *c* dimension at time step *t*.

#### 3.1.4 Mask generation.

The mask probabilities are generated based on the probability distribution of the final motion information $x_{\text{fin\_mot}}$. First, the normalized motion information $x_{\text{norm\_mot}}\in {\mathbb{R}}^{1\times T\times V\times C}$ is obtained by dividing the final motion information $x_{\text{fin\_mot}}$ by the maximum value in this time series *T*.

$${x_{\text{norm\_mot}}}_{v}^{t}(c)=\frac{{x_{\text{fin\_mot}}}_{v}^{t}(c)}{\max_{t^{\prime }}\left({x_{\text{fin\_mot}}}_{v}^{t^{\prime }}(c)\right)+1e-10}$$ (4)

where $\max_{t^{\prime }}\left({x_{\text{fin\_mot}}}_{v}^{t^{\prime }}(c)\right)$ denotes the maximum value on *T* at the time step $t^{\prime }$, and the ${x_{\text{norm\_mot}}}_{v}^{t}(c)$ denotes the normalized motion information of joint *v* in the *c* dimension at time step *t*.

Next, softmax is used to convert the normalized motion information $\hat{x}=\text{Decoder\_Pred}\left(x_{\text{dec }}\right)$ into mask probabilities.

$${x_{\text{mot\_prob}}}_{v}^{t}(c)=\mathrm{Softmax}\left(\frac{{x_{\text{norm\_mot}}}_{v}^{t}(c)}{\varphi }\right)$$ (5)

Here,

- φ = Temperature coefficient. Specifically, the value of φ controls the smoothness of the mask probability distribution. A higher temperature makes the distribution flatter, leading to more random masking choices, while a lower temperature concentrates the distribution, making the masking choices more focused. The value of φ is set to 0.8 in the experiments, selected by empirical tuning, with the goal of balancing randomness and focus in the masking process.

where ${x_{\text{mot\_prob}}}_{v}^{t}(c)$ denotes the mask probability of joint *v* in the *c* dimension at time step *t*.

To prevent any single dimension from excessively influencing the mask generation, the probability vector of the joint at each time step is averaged, and the resulting value is used as the final probability for that joint, denoted as $x_{\text{fin\_prob}}\in {\mathbb{R}}^{T\times V}$.

$${x_{\text{fin\_prob}}}_{v}^{t}=\frac{\sum_{c}{x_{\text{mot\_prob}}}_{v}^{t}(c)}{C}$$ (6)

where ${x_{\text{fin\_prob}}}_{v}^{t}$ denotes the probability that joint *v* is masked at time step *t*.

Next, dynamic mask generation is achieved by sampling the probability distribution $x_{\text{fin\_prob}}$ with the Gumbel-Softmax technique to determine which time steps are masked during each training epoch.

$$noise=\log\left(x_{\text{fin\_prob}}\right)-\log(-\log(\text{rand}))$$ (7)

Here,

- rand∈[0,1] = Random noise.

where $noise\in {\mathbb{R}}^{T\times V}$ denotes the mask probability matrix with noise. The ordering standard is the noise values of the joints in *noise* in both dimensions at each time step. The minimum sorting of the noise value at the time step is selected to obtain the sorting index of the time step *index*.

Furthermore, based on *index* and mask_ratio, this step select the first len_t time steps to keep, i.e., time steps with higher probability are selected to be kept, and time steps with lower probability are masked out.

$$len\_t=\left\lceil L^{*}(1-mask\_ratio)\right\rceil$$ (8)

Here,

- *L* = Total number of time steps.

where mask_ratio is the proportion of masks that take values between 0 and 1.

Ultimately, a binary mask matrix $mask\in {\mathbb{R}}^{1\times T\times V}$ is generated to indicate whether each time step is preserved or not.

$$mask_{t}^{v}=\left\{ \begin{array}{cc} 1, & \text{ if t is kept }\\ 0, & \text{ otherwise } \end{array}\right.$$ (9)

where $mask_{t}^{v}$ denotes whether joint *v* is masked at time step *t*. If the value is 1, it is masked, otherwise, it is not.

### 3.2 Topology-guided transformer model

**Fig 4:**
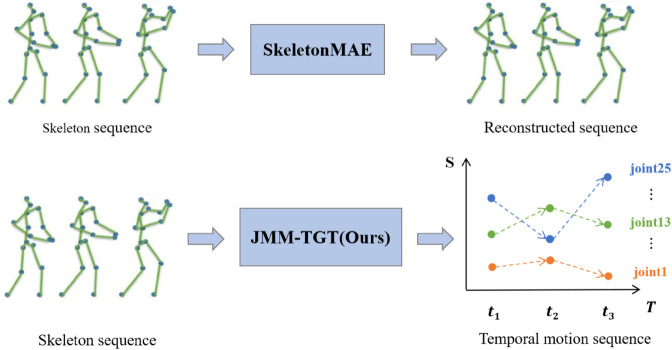
Comparison of pre-training objectives with SkeletonMAE(ICMEW 23) [12].

A detailed explanation of the data flow for the raw skeleton sequence in the JMM-TGT model is provided, highlighting the key design of the topology-guided transformer encoder. The overall processing flow is as follows: the pre-processed skeleton data is first converted into feature embeddings by using convolution operations, and then combined with positional and temporal encoding to help the model understand the relative positions and timing relationships of the joints. Next, a mask matrix is generated by using a joint motion-based masking strategy and applied to the skeleton data for masking. After the masking process is complete, the masked skeleton data is passed to the encoder, where the model learning spatio-temporal dependencies within the skeleton sequences through the multilayer topology-guided transformer. Finally, as shown in Fig 4, the encoder output is passed to the decoder for prediction of the temporal motion skeleton sequence.

#### 3.2.1 Embedding.

First, the preprocessed skeleton data is converted to feature embedding. The convolution kernel size of $\text{ Kernel Size }=\left[t_{\text{patch }},\text{patch\_size}\right]$ is used for the convolution operation.

$$x_{\text{emb}}=Conv2d(x)$$ (10)

In this way, embedding $x_{\text{emb }}\in {\mathbb{R}}^{N\times \left(T-t_{\text{patch }}+1\right)\times (V-\text{patch\_size}+1)\times (\text{dim\_feat})}$ is obtained. Next, positional and temporal coding is added to help the model understand the relative positions of the skeleton joints as well as the temporal relationships between frames.

$$x_{\text{temp }}=x_{\text{emb }}+{𝐄}_{𝐭}$$ (11)

$$x_{\text{pos }}=x_{\text{temp }}+{𝐄}_{𝐬}$$ (12)

Here,

- ${𝐄}_{𝐭}$ = Temporal coding.
- ${𝐄}_{𝐬}$ = Positional coding.

where $x_{\text{temp }}$ denotes the skeleton data obtained by adding temporal encoding to $x_{\text{emb }}$, $x_{\text{pos }}$denotes the skeleton data obtained by adding positional encoding to $x_{\text{temp }}$.

#### 3.2.2 Masking.

Next, the JMM strategy in Sect 3.1 obtains a mask matrix *mask* for the skeleton data $x\in R^{N\times T\times V\times C}$, which is then used to mask *x*_*pos*_.

$$I\left({\hat{y}}_{i}=y_{i}\right)$$ (13)

$$x_{\text{masked }}=\mathrm{Masking}\left(x_{\text{pos}},\text{mask}\right)$$ (14)

Here,

- JMM = Our joint motion masking strategy.

where $x_{\text{masked}}$ is the masked skeleton data, and Masking refers to the process of masking the time steps of certain joints in $x_{\text{pos}}$ using the mask matrix *mask*.

#### 3.2.3 Topology-guided transformer encoder.

The topology-guided transformer (TGT) incorporates the topological relationships between skeleton joints into the attention mechanism to optimize the calculation of attention scores. These relationships are represented using an adjacency matrix, which is introduced as prior knowledge. This allows the attention mechanism to better model interactions between connected joints, enhancing the ability of model to learn spatial dependencies within the skeleton data.

**Fig 5:**
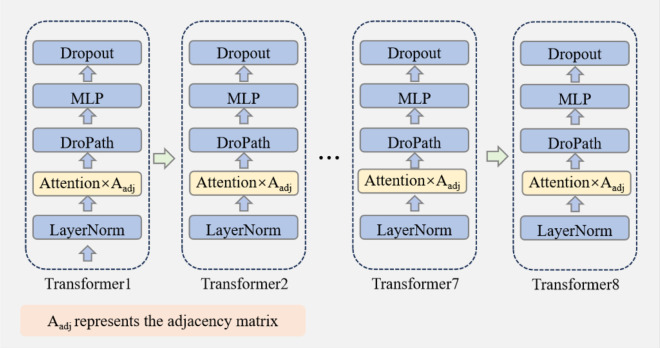
Architecture of topology-guided transformer encoder.

The encoder consists of eight transformer blocks, each containing a LayerNorm layer, an Attention layer, a DropPath layer, an MLP layer and a Dropout layer, as shown in Fig 5 below. Each layer serves a specific function to help the model learn complex spatio-temporal dependencies. The LayerNorm layer enhances training stability and prevents gradient issues, such as vanishing or exploding, by normalizing the inputs. The Attention layer adjusts attention scores using the adjacency matrix, modeling the interactions between joints to effectively capture long-range dependencies. The DropPath layer acts as regularization, helping the model avoid overfitting. The MLP layer strengthens the expressive power of the model through nonlinear transformations. Moreover, dropout improves the generalization ability of the model during training by randomly dropping neurons.

First, the attention score is calculated.

$$A=\text{Softmax}\left(x_{\text{masked }}\right)$$ (15)

Here,

- *A* = Attention score.

To strengthen spatial structure modeling, the adjacency matrix of 25 skeleton joints is used in the calculation of attention scores. This matrix is predefined based on the topological structure of the human skeleton, encoding the relationships between joints and remaining fixed during training. The adjacency matrix *A*_*adj*_ is represented as:

$$A_{ij}=\left\{ \begin{array}{cc} 1, & v_{i}\text{ and }v_{j}\text{ are connected }\\ 0, & v_{i}\text{ and }v_{j}\text{ are not connected } \end{array}\right.$$ (16)

where *A*_*ij*_ denotes the connection relationship between joint $v_{i}$ and joint $v_{j}$.

The adjusted attention score is:

$$A^{\prime }=A\times A_{adj}$$ (17)

Here,

- *A*_*adj*_ = Adjacency matrix. It serves as prior in the calculation of the attention layer.
- $A^{\prime }$ =Adjusted attention score.

The output of the Attention layer is then passed as input to the DropPath layer and continues through the network. After eight transformer blocks, the final output of the encoder is denoted as *x*_*enc*_.

$$x_{\text{enc }}=\mathrm{Encoder}\left(x_{\text{masked }}\right)$$ (18)

#### 3.2.4 Decoder.

**Fig 6:**
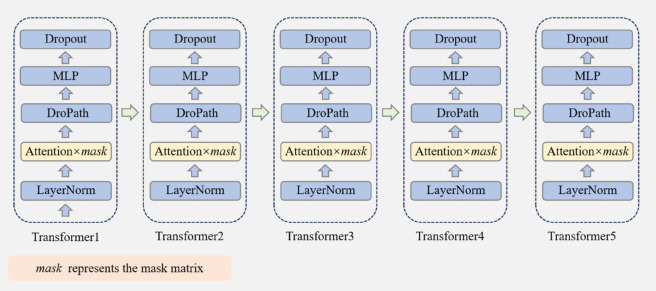
Architecture of transformer-based decoder.

The task of the decoder is to reconstruct the skeleton data for the masked time steps based on the output from the encoder. Decoder operates similarly to the encoder, except that the Attention layer utilizes a mask matrix to adjust the attention scores, as shown in Fig 6 below. After passing through five transformer layers, the final output of the encoder is denoted as $\hat{x}$.

$$A^{\prime }=A\times mask$$ (19)

$$x_{\text{dec }}=\mathrm{Decoder}\left(x_{\text{enc }}\right)$$ (20)

$$\hat{x}=\text{Decoder\_Pred}\left(x_{\text{dec }}\right)$$ (21)

where $A^{\prime }$ denotes the attention score adjusted by the mask matrix *mask*, which is used in the attention layer, and *x*_*dec*_ is the decoder intermediate input after the multilayer transformer module. Decoder_Pred refers to the prediction layer of the decoder.

#### 3.2.5 Loss function.

Finally, the loss function computes the reconstruction error between the predicted skeleton sequence $\hat{x}$ and the original skeleton sequence *x*. Mean squared error is used here.

$$L=\frac{1}{N}\sum_{i=1}^{N}\sum_{t=1}^{T}\sum_{v=1}^{V}{\left({\hat{x}}_{i,t,v}-x_{i,t,v}\right)}^{2}\cdot mask$$ (22)

Here,

- *mask* = Binary mask matrix.
- $\hat{x}$ =Reconstruction skeleton data.
- *N* =Number of samples.
- L =Reconstruction error.

## 4 Experiment

This section presents comparative experiments between the JMM-TGT proposed in this paper and other mainstream models on the NTU RGB+D 60, NTU RGB+D 120, and PUK-MMD datasets, along with the results and analysis of the ablation experiments.

**Dataset and dataset preprocessing.**

The experimental datasets in this paper is based on the publicly available NTU RGB+D 60 (NTU-60) dataset [43], NTU RGB+D 120 (NTU-120) dataset [44], and PKU-MMD dataset [45]. The NTU-60 dataset contains 60 action categories captured from 40 different subjects in a multi-camera setup, with a total of 56,880 samples. The NTU-120 dataset extends the NTU-60 dataset, adding 120 action categories and a total of 113,945 samples. The PKU-MMD dataset contains nearly 20,000 action instances, over 5 million frames, and covers 51 action categories.

To better evaluate the robustness and generalization ability of the model, datasets under different protocols are used, accounting for variations in shooting angles and subject differences. Specifically, the Cross-Subject (X-Sub) and Cross-View (X-View) protocols are used for NTU-60, and the Cross-Subject (X-Set) and Cross-View (X-View) protocols are used for NTU-120. The PKU-MMD includes two subsets, the first part (PKU-I) and the second part (PKU-II). Compared to PKU-I, the PKU-II is more challenging due to its more complex viewpoints. PKU-I and PKU-II are divided into training and test sets according to a cross-subject protocol.

Before feeding the data into the model, the original action videos are preprocessed by unifying them to a fixed length of 120 seconds through random sampling, maximizing training data diversity. Due to the video being too long, each input video is further divided into four smaller segments [12] to reduce server response time. The input skeleton data consists of 25 joints, each with three dimensions.

**Experimental setup.**

In the topology-guided transformer module, the encoder depth is set to 8, the decoder depth to 5, and the transformer feature dimension per layer is 256. The JMM-TGT model is trained for 400 epochs with a batch size of 128, using the AdamW optimizer with a weight decay of 0.05. The learning rate follows a warm-up strategy of 20 epochs, increasing linearly from 0 to 1e-3, and then decreasing to 1e-4 by using a cosine decay. In the linear probing evaluation protocol, the pre-trained parameters are fixed, and a linear classifier is added after JMM-TGT. The model is trained for 100 epochs with a batch size of 48 and a learning rate of 0.1.

In the fine-tuning evaluation protocol, an MLP layer is added after the pre-trained JMM-TGT and trained for 100 epochs with a batch size of 32. The learning rate is linearly increased from 0 to 2e-4, then reduced to 1e-5 using a cosine decay scheme.

In the semi-supervised evaluation protocol, a classification layer is added after the pre-trained JMM-TGT, and the model is fine-tuned on a small training set. Other settings remain consistent with the fine-tuning protocol.

In the transfer learning evaluation protocol, the pre-trained JMM-TGT is connected with a linear classifier and fine-tuned on the target dataset. The training epochs, batch size, and learning rate are consistent with the settings used in the fine-tuning evaluation protocol. Finally, all experiments are conducted on three NVIDIA RTX 3090 Ti using the PyTorch framework.

**Evaluation metrics.**

This paper primarily uses Top-1 accuracy as the evaluation metric to measure the final classification performance of the JMM-TGT model. Top-1 accuracy measures whether the most probable category predicted by the model matches the true label. This metric is especially effective in 3D skeleton action recognition, as it reflects the ability of model to correctly classify actions. The formula for calculating Top-1 accuracy is:

$$\text{ Top-1 }=\frac{1}{N}\sum_{i=1}^{N}I\left({\hat{y}}_{i}=y_{i}\right)$$ (23)

Here,

- *N* =Number of test samples.
- ${\hat{y}}_{i}$ =Predicted label of sample *i*.
- *y*_*i*_= True label of sample *i*.
- $I\left({\hat{y}}_{i}=y_{i}\right)$ = Indicator function.

### 4.1 Comparison experiment

JMM-TGT is evaluated under four evaluation protocols: linear probing evaluation protocol, fine-tuning evaluation protocol, semi-supervised evaluation protocol, and transfer learning evaluation protocol. The linear probing evaluation protocol evaluates the quality of the learned feature representations by training a simple linear classifier on top of the pre-trained model. The fine-tuning evaluation protocol, on the other hand, is designed to evaluate the performance of model on downstream tasks. A comparative analysis with supervised learning methods is also conducted. The semi-supervised evaluation protocol combines a limited amount of labeled data with a large volume of unlabeled data to evaluate the ability of the model to generalize with minimal labeled data. Finally, the transfer learning evaluation protocol aims to assess whether the representations learned by the pre-trained model on the source dataset can be successfully transferred and applied to the target dataset, testing the generalization ability of model.

**Fig 7:**
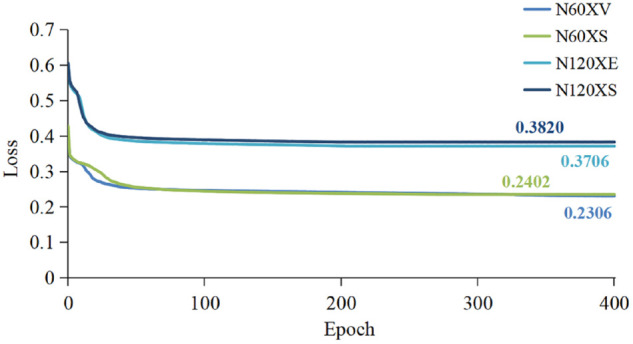
Pre-training loss of the JMM-TGT model on the NTU-60 and NTU-120 datasets.

Before these protocols, the pre-training loss of the JMM-TGT model is shown over 400 epochs on the NTU-60 and NTU-120 datasets. As shown in Fig 7, the loss gradually decreases and stabilizes with increasing training epochs. This indicates that the JMM-TGT model is gradually converging during training and its performance is steadily improving, thus making thorough preparations for the subsequent evaluation stages.

#### 4.1.1 Linear probing evaluation experiment.

The effectiveness of JMM-TGT in feature learning is validated by comparing it with state-of-the-art self-supervised and unsupervised learning methods on the NTU-60 and NTU-120 datasets. These state-of-the-art methods include those based on masking strategy, such as SkeletonMAE [12], as well as those based on contrastive learning, including ActCLR [29], SSRL [34], 3s-SSRL [34], DMMG [38], SG-CLR [42], AimCLR [46], 3s-AimCLR [46], GT-Transformer [47], CPM [48], CMD [49], and HiCo-Transformer [50].

As shown in Table 2, JMM-TGT outperforms the contrastive learning-based methods and also surpasses SkeletonMAE [12], across different protocols of the two datasets. Notably, JMM-TGT uses only a single joint stream as input. Meanwhile, JMM-TGT achieves impressive results on the X-Sub and X-View protocols of NTU-60, with accuracies of 84.4% and 90.1%, respectively. It outperforms the recent method SG-CLR [42] by 0.9% and 0.2%, respectively. This demonstrates that the joint motion-based masking strategy and the topology-guided transformer help the encoder better learn to discriminate and represent skeleton features.

**Table 2:** Comparison of linear probing evaluation results with state-of-the-art methods on the NTU-60 and NTU-120 datasets.

Methods	Stream	NTU-60(%)		NTU-120(%)	
		X-Sub	X-View	X-Sub	X-Set
3s-AimCLR(AAAI 22) [46]	J+B+M	78.9	83.8	68.2	68.8
3s-SSRL(IEEE TCSVT 23) [34]	J+B+M	81.6	85.1	69.2	71.5
AimCLR(AAAI 22) [46]	J	74.3	79.7	63.4	63.4
SkeletonMAE(ICMEW 23) [12]	J	74.8	77.7	72.5	73.5
GT-Transformer(ECVC 22) [47]	J	76.3	83.8	66.0	68.7
CPM(ECVC 22) [48]	J	78.7	84.9	68.7	69.6
CMD(ECVC 22) [49]	J	79.4	86.9	70.3	71.5
SSRL(IEEE TCSVT 23) [34]	J	80.4	82.0	68.0	68.6
ActCLR(CVPR 23) [29]	J	80.9	86.7	69.0	70.5
HiCo-Transformer(AAAI 23) [50]	J	81.1	88.6	72.8	74.1
DMMG(IEEE T Image Process 23) [38]	J	82.1	87.1	69.9	70.1
SG-CLR(Pattern Recogn 25) [42]	J	83.5	89.9	75.3	77.1
**JMM-TGT (Ours)**	**J**	**84.4**	**90.1**	**78.2**	**78.8**

J, M, and B denote joint, motor, and skeletal flows, respectively. J+B+M implies a fusion of the three flows.

On the X-Sub and X-Set protocols of NTU-120, JMM-TGT achieves 78.2% and 78.8% accuracy, outperforming existing methods. Compared to SkeletonMAE [12], JMM-TGT improves by 5.7% and 5.3% on the X-Sub and X-Set protocols, respectively. Meanwhile, JMM-TGT surpasses the state-of-the-art SG-CLR [42] method, leading by 2.9% and 1.7% in the X-Sub and X-Set protocols, respectively. This shows that JMM-TGT is highly competitive even with large datasets and multiple action categories.

#### 4.1.2 Fine-tuning evaluation experiment.

The fine-tuning performance of JMM-TGT is evaluated on the NTU-60 and NTU-120 datasets. Similarly, comparisons are made with the latest methods, including SkeletonMAE [12], including SSRL [34], 3s-SSRL [34], SG-CLR [42], 3s-SG-CLR [42], AimCLR [46], 3s-AimCLR [46], CPM [48], and 3s-Hi-TRS [51].

As shown in Table 3, the JMM-TGT achieves accuracies of 92.2% and 96.9% for X-Sub and X-View protocols of NTU-60, respectively. Comparison to SkeletonMAE [12], it improves by 5.6% and 4.0%, respectively. In addition, compared to the multi-stream 3s-Hi-TRS [51], which also employs transformer, it outperforms by 2.2% and 1.2%, respectively. Moreover, on the X-Sub and X-Set protocols of NTU-120, JMM-TGT achieves an accuracy of 89.7% and 90.5% after fine-tuning. To be specific, compared to the multi-stream 3s-Hi-TRS [51], it surpasses 3s-Hi-TRS [51] by 4.4% and 3.1%, respectively.

**Table 3:** Comparison of fine-tuning evaluation results with state-of-the-art methods on the NTU-60 and NTU-120 datasets.

Methods	Network	NTU-60(%)		NTU-rlap120(%)	
		X-Sub	X-View	X-Sub	X-Set
SSRL(IEEE TCSVT 23) [34]	GCN	83.2	90.3	76.5	77.2
3s-SSRL(IEEE TCSVT 23) [34]	GCN	87.0	93.0	80.3	81.7
AimCLR(AAAI 22) [46]	ST-GCN	83.0	89.2	77.2	76.0
SG-CLR(Pattern Recogn 25) [42]	ST-GCN	83.8	91.4	75.6	76.3
CPM(ECCV 22) [48]	ST-GCN	84.4	91.1	78.4	78.9
3s-AimCLR(AAAI 22) [46]	ST-GCN	86.9	92.8	80.1	80.9
3s-SG-CLR(Pattern Recogn 25) [42]	ST-GCN	88.2	94.1	83.0	83.6
AimCLR(AAAI 22) [46]	STTFormer	83.9	90.4	74.6	77.2
SkeletonMAE(ICMEW 23) [12]	STTFormer	86.6	92.9	76.8	79.1
3s-Hi-TRS(ECCV 22) [51]	Transformer	90.0	95.7	85.3	87.4
**JMM-TGT (Ours)**	**Transformer**	**92.2**	**96.9**	**89.7**	**90.5**

Overall, JMM-TGT shows significant performance improvements on the NTU-60 and NTU-120 datasets. The final result outperforms all previous methods, even those with multi-stream fusion, such as 3s-SSRL [34], 3s-SG-CLR [42], 3s-AimCLR [46], and 3s-Hi-TRS [51]. In addition, JMM-TGT performs notably better than GCN, ST-GCN, and STTFormer, further validating the effectiveness of the transformer in action recognition tasks.

Moreover, the JMM-TGT is compared with top-performing supervised methods like ST-GCN [6], Shift-GCN [15], SAN-GCN [16], and MSS-GCN [17]. As shown in Table 4, JMM-TGT significantly outperforms supervised methods such as ST-GCN [6], Shift-GCN [15], and SAN-GCN [16] on both the NTU-60 and NTU-120 datasets. In the X-View protocol of NTU-60, JMM-TGT achieves a top accuracy of 96.9%, tying with the MSS-GCN [17]. In the X-Sub protocol of NTU-120, JMM-TGT leads with the highest accuracy of 89.7%, its accuracy is just 0.1% lower than that of MSS-GCN [17]. These results demonstrate that JMM-TGT excels on both benchmark datasets and remains highly competitive with state-of-the-art supervised learning methods.

**Table 4:** Comparison with state-of-the-art supervised methods on the NTU-60 and NTU-120 datasets.

Methods	NTU-60(%)		NTU-120(%)	
	X-Sub	X-View	X-Sub	X-Set
ST-GCN(AAAI 18) [6]	81.5	88.3	72.4	71.3
Shift-GCN(CVPR 20) [15]	89.7	96.0	85.3	86.6
SAN-GCN(IEEE TMM 23) [16]	92.1	96.2	88.7	90.1
MSS-GCN(IEEE TCSVT 24) [17]	**92.7**	96.9	88.9	**90.6**
**JMM-TGT (Ours)**	92.2	**96.9**	**89.7**	90.5

#### 4.1.3 Semi-supervised evaluation experiment.

In semi-supervised learning, the performance is reported on the NTU-60 dataset using 1% and 10% of the labeled training data. Comparing with state-of-the-art methods, including 3s-SSRL [34], 3s-SG-CLR [42], 3s-AimCLR [46], GT-Transformer [47], CPM [48], CMD [49], HiCo-Transformer [50], LongT GAN [52], and M${\text{S}}^{2}$L [53].

As shown in Table 5, even with only 1% labeled data, JMM-TGT achieves an accuracy of 65.3% and 67.5% on X-Sub and X-View protocols, respectively. With only 1% labeled data, JMM-TGT outperforms the state-of-the-art methods in all settings. Specifically, JMM-TGT surpasses 3s-SSRL [34] by 4.1% and 11.2% in X-Sub and X-View protocols of NTU-60, respectively.

**Table 5:** Comparison of semi-supervised evaluation results with state-of-the-art methods on the NTU-60 dataset.

Method	NTU-60(%)			
	X-Sub		X-View	
	(1%)	(10%)	(1%)	(10%)
LongT GAN(AAAI 18) [52]	35.2	62.0	-	-
M${\text{S}}^{2}$L(ACM MM 20) [53]	33.1	65.2	-	-
GT-Transformer(ECCV 22) [47]	-	68.6	-	74.9
CMD(ECCV 22) [49]	50.6	75.4	53.0	80.2
HiCo-Transformer(AAAI 23) [50]	54.4	73.0	54.8	78.3
3s-AimCLR(AAAI 22) [46]	54.8	78.2	54.3	81.6
3s-SG-CLR(Pattern Recogn 25) [42]	56.0	81.3	56.5	84.6
CPM(ECCV 22) [48]	56.7	73.0	57.5	77.1
3s-SSRL(IEEE TCSVT 23) [34]	61.2	79.4	56.3	82.0
**JMM-TGT (Ours)**	**65.3**	**87.3**	**67.5**	**89.2**

The performance of JMM-TGT model further improves to 87.3% and 89.2% when 10% labeled data is available. Notably, when using just 10% labeled data, JMM-TGT shows even greater improvements over other methods, outperforming 3s-SSRL [34] by 7.9% and 7.2% on X-Sub and X-View protocols, respectively. This demonstrates the capability of JMM-TGT in extracting more discriminative skeleton representations.

#### 4.1.4 Transfer learning evaluation protocol experiment.

In the transfer learning, NTU-60, NTU-120, and PKU-I are selected as the source datasets, with PKU-II chosen as the target dataset. Comparing with state-of-the-art methods, including LongT GAN [52], M${\text{S}}^{2}$L [53], ICS [54], and CMD [49].

As shown in Table 6, the proposed JMM-TGT achieves the best performance on the PKU-II dataset, indicating that the representations learned by the method have stronger transferability. On the source datasets NTU-60, NTU-120, and PKU-I, JMM-TGT outperforms LongT GAN [52], M${\text{S}}^{2}$L [53], ICS [54], and CMD [49] to varying degrees. Specifically, when pretraining on the NTU-60 and NTU-120 datasets, it improves by 15.4% and 16% over CMD [49], respectively. When pretraining on the PKU-I dataset, it outperforms ICS [54] by 25.5%. These results confirm the excellent generalization ability of the representations learned by the JMM-TGT.

**Table 6:** Comparison of transfer learning protocol results with state-of-the-art methods on the PKU-II dataset.

Models	Transfer learning to PKU-II		
	NTU-60(%)	NTU-120(%)	PKU-I(%)
LongT GAN(AAAI 18) [52]	44.8	-	43.6
M${\text{S}}^{2}$L(ACM MM 20) [53]	45.8	-	44.1
ICS(ACM MM 21) [54]	51.1	52.3	45.1
CMD(ECCV 22) [49]	56.0	57.0	-
**JMM-TGT(Ours)**	**71.4**	**73.0**	**70.6**

### 4.2 Ablation experiment

Ablation studies are conducted to validate the contribution of the different components of JMM-TGT to the overall performance. All experimental results focus on linear probing evaluation protocol, using the X-Sub protocol on the NTU-60 dataset.

#### 4.2.1 Effectiveness of JMM-TGT.

To evaluate the effectiveness of the joint motion masking strategy and the topology-guided transformer, this study explores how the three components (that is to say, joint motion difference, motion similarity, and topology information) impact the accuracy of 3D skeleton action recognition. This paper use two baseline models: one that considers only joint motion difference (Baseline-1) and one that considers only joint motion similarity (Baseline-2). JMM-TGT* denotes the model with the topology-guided component removed.

As shown in Table 7, under the linear probing evaluation protocol, Baseline-1 and Baseline-2 achieve accuracies of 82.2% and 82.4%, respectively, demonstrating the effectiveness of each component in isolation. When joint motion difference and motion similarity are combined, JMM-TGT* sees an accuracy boost of 1.6% and 1.4% over Baseline-1 and Baseline-2, respectively. This suggests an interaction between the two components. When all components are used together, the accuracy reaches 84.4%, showing a significant synergistic effect of joint motion difference, motion similarity, and topology information in improving overall model performance.

**Table 7:** Ablation experiments of JMM-TGT components and complexity comparison with state-of-the-art methods on the NTU-60 dataset.

Models	JM difference	JM similarity	Topology guided	Params (M)	FLOPs (G)	Runtime (s)	NTU-60 (%)
AimCLR(AAAI 22) [46]	-	-	-	1.15	0.84	4	74.3
HiCo-Transformer(AAAI 23) [50]	-	-	-	3.87	0.77	4	81.1
Baseline-1	√	-	-	7.97	3.56	14	82.2
Baseline-2	-	√	-	7.97	3.56	14	82.4
JMM-TGT*	√	√	-	7.97	3.56	14	83.8
**JMM-TGT(Ours)**	√	√	√	**7.97**	**3.56**	14	**84.4**
DMMG*(IEEE TIP 23) [38]	-	-	-	28.72	25.7	107	82.1
ST-GCN(AAAI 18) [6]	-	-	-	24.34	16.2	67	81.5
ICS(ACM MM 21) [54]	-	-	-	45	5.76	24	76.3
SkeletonMAE(ICMEW 23) [12]	-	-	-	11	-	20	74.8

JM denotes joint motion, Runtime denotes the training time per epoch, DMMG* represents our own implementation. All the results are evaluated in the same environment using one RTX 3090ti GPU.

In addition, the performance of the JMM-TGT model is evaluated in terms of training time, parameter count, and FLOPs. Overall, JMM-TGT is a mid-scale model with relatively low computational complexity. Each training epoch takes 14 seconds, which is longer than HiCo-Transformer[50]. This difference is primarily due to the additional joint motion difference and similarity calculation modules introduced in JMM-TGT, which significantly enhance the representational power of the model. JMM-TGT has 7.97M parameters and 3.56G FLOPS, both of which are considerably lower than those of DMMG*[38] and ST-GCN [6], thereby striking an effective balance between computational efficiency and model expressiveness. Ultimately, JMM-TGT achieved an accuracy of 84.4% on the X-Sub protocol of NTU-60, surpassing lightweight models such as HiCo-Transformer [50] and AimCLR [46]. This demonstrates that the additional computational cost results in significant performance improvements. The above results suggest that JMM-TGT effectively balances computational cost, model complexity, and performance.

#### 4.2.2 Effectiveness of masking strategy.

To further investigate the effectiveness of the joint motion masking strategy, the joint motion masking strategy in JMM-TGT is replaced with random masking in the experiments. In addition, the impact of the masking ratio on the experiments is explored.

As shown in Table 8, compared to the random masking strategy, our joint motion masking strategy improves the absolute performance on NTU-60 dataset by 1.5%. This demonstrates that combining joint motion difference and similarity provides a rich semantic prior, effectively guiding the skeleton masking process.

**Table 8:** Ablation experiments with masking strategy on linear probing evaluation protocol.

Methods	NTU-60(%)
Random masking	82.9
**Joint motion masking (Ours JMM)**	**84.4**

**Fig 8:**
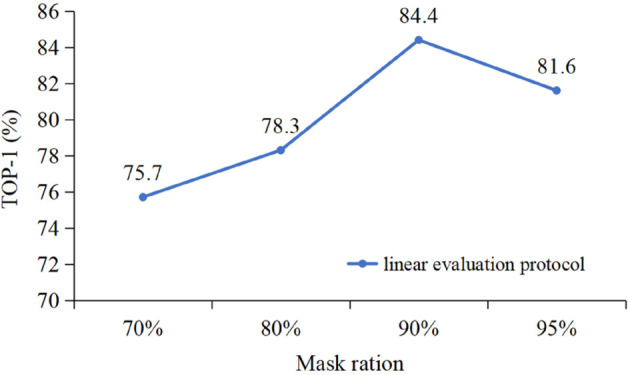
Ablation experiment for mask ratio.

As shown in Fig 8, the effect of different masking ratios on the final experimental results is evaluated. With a 70% masking ratio, the accuracy is 75.7%. As the masking ratio increases, the accuracy improves, reaching 84.4% at 90%. However, when the masking ratio is increased to 95%, the accuracy drops to 81.6%. Results on the X-Sub protocol indicate that a 90% masking ratio provides optimal performance for the JMM-TGT.

Therefore, choosing the appropriate masking ratio s crucial for balancing model performance. Empirical findings suggest that both excessively high and low masking ratios can negatively affect results. A low ratio may prevent the model from capturing enough joint motion variations, while a high ratio can smooth out too much data, losing important motion details. Based on our findings, a 90% masking ratio strikes the best balance between data masking and model accuracy. For different datasets, the masking ratio can be adjusted between 80% and 95%, based on its impact on model performance.

## 5 Visualization

**Fig 9:**
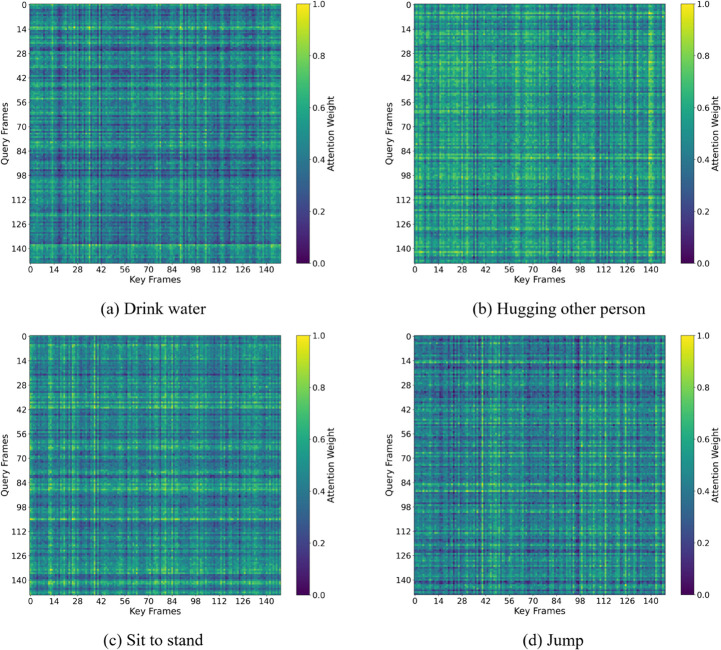
Visualization of attention weight distribution on the NTU-60 dataset.

To visually demonstrate the effectiveness of the JMM-TGT, attention weight heatmaps are used to analyze the temporal relationships and focus of attention in action recognition results. As shown in Fig 9, the time attention weight distribution of the first attention head in the last layer for four action types is extracted. In the attention heatmap, the color green, from dark to light, indicates the attention weight ranging from low to high.

For complex actions like hugging, the attention weight is spread across multiple frames, indicating that the JMM-TGT captures details across a wider range to handle higher complexity. In contrast, for simpler actions like drinking, the attention is focused on a few key frames, avoiding redundant information. For fast movements like jumping, the model focuses on frames with significant changes, demonstrating its sensitivity to dynamic variations. The results show that the JMM-TGT model, by introducing a topology-guided attention mechanism and incorporating spatial dependencies between joints, can more accurately understand the spatiotemporal evolution of human actions.

## 6 Conclusion

In this work, we propose the JMM-TGT model for self-supervised 3D skeleton action recognition. The model introduces a joint motion masking strategy and a topology-guided transformer, which effectively fuse global and local spatio-temporal features, significantly improving model performance in complex skeleton action recognition tasks. Specifically, the JMM integrates the differences and similarities in joint motion across adjacent frames and each dimension as the final motion information. The information is then transformed into a probability distribution for the mask, which dynamically guides the selection of joints to mask at each time step. The method captures the dynamic changes of each joint and effectively guide the model in selecting critical time-step information during training. The TGT adjusts the attention mechanism based on the topological relationships between joints, which ensures the model focuses on spatio-temporal interactions when learning skeleton sequences, and further enables the understanding of complex actions. Extensive experiments are conducted on three popular benchmarks using four evaluation protocols. The results show that the JMM-TGT model achieves state-of-the-art performance on the NTU-60 and NTU-120 datasets, validating the efficiency of the skeleton representation and enhancing the potential of the transformer in action recognition. Moreover, the model demonstrates strong generalization when applied to the PKU-MMD dataset, showcasing its robustness for real-world applications. Additionally, JMM-TGT achieves low computational complexity, balancing computational efficiency with accuracy.
